# Normative amygdala fMRI response during emotional processing as a trait of depressive symptoms in the UK Biobank

**DOI:** 10.1017/S0033291725101797

**Published:** 2025-10-08

**Authors:** Jerke J. van den Berg, Henricus G. Ruhé, Henk A. Marquering, Liesbeth Reneman, Matthan W. A. Caan

**Affiliations:** 1 Biomedical Engineering and Physics, Amsterdam UMC Location AMC, University of Amsterdam, Amsterdam, The Netherlands; 2 Radiology and Nuclear Medicine, Amsterdam UMC Location AMC, University of Amsterdam, Amsterdam, The Netherlands; 3 Department of Psychiatry, Radboud UMC, Nijmegen, The Netherlands; 4 Donders Institute for Brain, Cognition and Behavior, Radboud University, The Netherlands

**Keywords:** Normative Modeling, Major Depressive Disorder, UK Biobank, Amygdala, Hariri Task, Neuroimaging

## Abstract

**Background:**

Heightened reactivity in the amygdala measured by functional magnetic resonance imaging during emotional processing is considered a potential biomarker for clinical depression. Still, it is unknown whether this is also true for depressive symptoms in the general population, and – when in remission after recurrent depressive episodes – it is associated with future episodes.

**Methods:**

Using the UK Biobank population study (*n* = 11,334), we investigated the association of amygdala reactivity during negative facial stimuli, focusing on lifetime depression (trait), depressive symptoms (state), and the modulating effect of antidepressant (AD) treatment thereof. We employed normative modeling (NM) to better incorporate population heterogeneity of the amygdala activity.

**Results:**

In line with a previous study, depressive symptoms (state) over the last 2 weeks were not associated with the amygdala reactivity signal. Rather, our results indicate a significant positive association (*p* = 0.03, *ω* ^2^ = 0.001) between amygdala response and the recurrence of depressive episodes (trait). Longitudinal analysis revealed that the group that had experienced a single depressive episode before showed a significantly increased amygdala response after additional episodes (*p* = 0.03, *ω* ^2^ = 0.017). ADs were not associated with amygdala response directly, but decreased associations within episode recurrence severity.

**Conclusions:**

The amygdala response to negative stimuli was associated with an individual’s risk of recurrence of depressive episodes, and AD treatment reduced these associations. This study highlights the relevance of amygdala reactivity as a trait, but not a state biomarker for (recurrent) depression. Moreover, it demonstrates the benefit of applying NM in the context of population data.

## Introduction

Depression is a common psychiatric disorder and a leading source of disability and disease burden worldwide (Zhang et al., 2024). Currently, diagnosing major depressive disorder (MDD) relies on the interpretation of clinical data and patient history by a clinician. There are no biological markers upon which to base treatment choices, resulting in treatments being applied by a trial-and-error process (K. M. Smith, Renshaw, & Bilello, 2013). By incorporating biological features, the diagnosis and prognosis of the disease may be further tailored to patients for a better-suited treatment of the disease (Dunlop & Mayberg, 2017). Functional neuroimaging may aid in increasing our understanding of MDD by linking the disorder to biological deviations and activity within brain regions. This knowledge may subsequently enhance the personalization of treatment.

Early brain imaging studies indicated that individuals with depression exhibit increased amygdala reactivity to negative faces compared to healthy controls (HCs) (Peluso et al., 2009; Siegle, Steinhauer, Thase, Stenger, & Carter, 2002). Recent meta-analyses, including the largest by Li and Wang (*n* = 2,383), consistently report increased functional magnetic resonance imaging (fMRI) measured amygdala activity in depressed patients assessed during emotional processing tasks involving facial stimuli (Li & Wang, 2021). This activity is measured in terms of the blood-oxygen-level-dependent (BOLD) contrast, which measures changes in blood oxygenation that occur in response to neuronal activity. Additionally, antidepressant (AD) treatment has been shown to decrease the amygdala BOLD signal, especially in treatment responders (Gerlach et al., 2022; Ruhé, Booij, Veltman, Michel, & Schene, 2012; Sheline et al., 2001). More recently, research has highlighted the persistently elevated amygdala activity to negative face stimuli over the course of the disease, even after remission (Klug et al., 2024). Thus, evidence from diverse research avenues supports the reciprocal association between depression and altered amygdala reactivity. However, prior research primarily focused on clinical MDD, and it is still unknown how emotional processing within the amygdala is associated with recurrent depressive symptoms in the general population. Moreover, more research in large datasets is needed to link amygdala reactivity with recurrent episodes of depression to better identify its association within the progression of MDD.

The population-based UK Biobank (UKB) is the largest publicly available emotional processing fMRI dataset (Sudlow et al., 2015). Tamm et al. recently investigated the association between emotional processing in the amygdala and depressive symptoms in the UKB and found – unexpectedly – no association (Tamm et al., 2022). The authors concluded that, in light of their work, it seems highly unlikely that emotional processing in the amygdala is of fundamental importance for the pathophysiology or symptomatology of depression. Their main analysis involved the correlation between depressive symptoms measured using the Recent Depressive Symptoms (RDS-4) questionnaire and amygdala reactivity (the faces–shapes contrast BOLD median masked to the amygdala region) from the first imaging visit. However, Tamm et al. did not correct for depressive symptom history and lifetime recurrence of episodes. The RDS-4 covers depressive *symptoms* and severity over 2 weeks before the day of scanning, and is largely based on the Patient Health Questionnaire (PHQ-9) (Dutt et al., 2022). Smith et al. previously validated the classification of a probable lifetime history of mood disorder in the UKB (D. J. Smith et al., 2013), based on the statistical prevalence of the disease in 172,751 participants. However, their classification did not fully account for extensive depressive episode recurrence further than two episodes (or more). One theory that has gained support in mental disorders is ‘Kindling’. This theory states that with an increase in major depressive episodes (MDEs), the relation between adverse life events and remission of depressive episodes weakens (Post, 1992). This implies that with an increasing number of MDEs experienced, the susceptibility to MDEs in the future increases, which might also result in the progression of recurrent depressive episodes to chronic episodes, currently captured in a comprehensive staging model (Hetrick et al., 2008). We, therefore, propose to extend the criteria set forward by Smith to lifetime experienced depressive episodes, as this adaptation may reveal relations not yet analyzed. Moreover, to accommodate the Kindling theory, in this study, we stage lifetime depression by modeling both depressive state and trait. By incorporating longitudinal data and correcting for AD treatment, the relationship between the occurrence of additional depressive episodes and amygdala signal (as a measure of the susceptibility to MDEs) may better be captured.

Normative modeling (NM) is a technique to capture normative trends within features on a population level over the span of additional explanatory variables, with the aim of mapping differences on the subject level. NM aids in accounting for heterogeneity present in the data by accounting for factors such as demographic variation. By reducing heterogeneity, NM may leverage the knowledge included in the large body of healthy participants present in the UKB, and increase the predictive value of biomarkers for mental and behavioral disorders in general (Marquand et al., 2019; Marquand, Rezek, Buitelaar, & Beckmann, 2016; Rutherford et al., 2023, 2022).

While applying NM, we aim to investigate the association between the amygdala response to negative faces (assessed by fMRI and modeled according to NM), and a clinically relevant classification of state and trait characteristics of MDD. Herein, we associate RDS-4 as well as the number of MDEs to amygdala response, hereby providing input on depressive state and trait, to quantify which characteristics relate to aberrant emotional processing. Additionally, we incorporate AD use to quantify the relation AD medication has on amygdala reactivity. We hypothesize that new episodes will increase amygdala activity, in accordance with the Kindling theory, but that ADs will dampen this signal, as observed in previous work (Gerlach et al., 2022; Ruhé et al., 2012; Sheline et al., 2001).

## Methods

### Data sample

**Figure 1.**
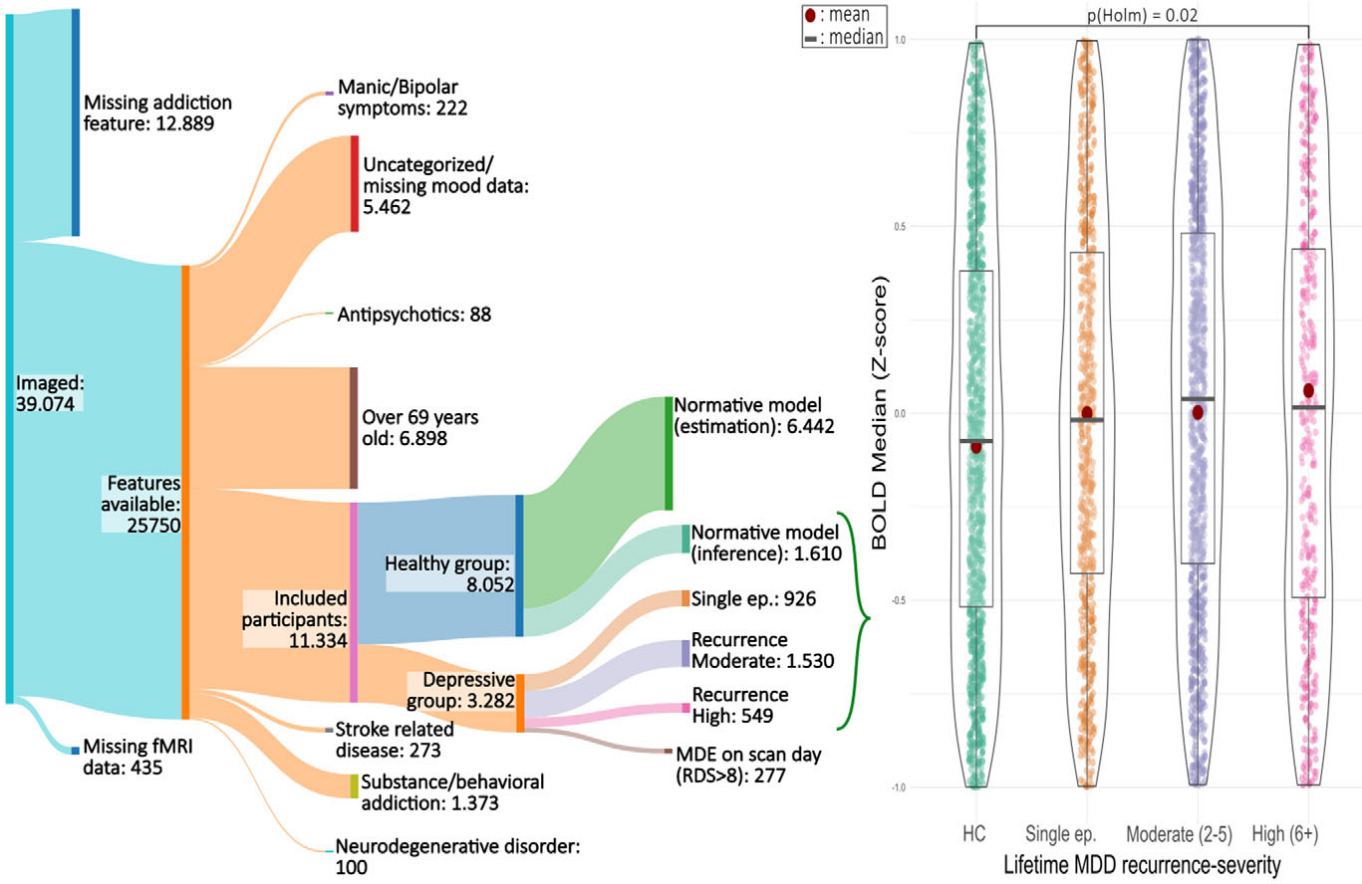
Flowchart of data exclusion criteria and the main cross-sectional (ANOVA) analysis (1a.1) performed on the normative model-derived median BOLD Z-scores in the amygdala per MDD recurrence severity (HC, single episode, moderate (two to five episodes), and high (≥6 episodes)), while in remission (RDS ≤ 8). *Y*-axis clipped at [−1, 1] for clarity. The top bar indicates significant post-hoc pairwise results. HC, healthy control.

The current investigation utilizes data derived from the UKB cohort, comprising adults drawn from the general population of the United Kingdom, with the recruitment period between 2006 and 2010 (Sudlow et al., 2015). Recruitment-targeted individuals were aged between 40 and 69 years, without additional exclusion criteria. After recruitment, three in-person follow-up visits were conducted among participants. The participation rate was ~5% for the initial imaging visit, which started in 2014. Participants eligible to attend the same site for a repeating imaging visit were invited back 24–27 months after their initial imaging visit. Around 12% attended a repeat imaging visit, on average 2.5 years after the first. A total of 6,442 HCs were used for fitting the NM, and 4,892 participants were analyzed across HC and symptomatic groups (see Figure 1). These two phases are analyzed in this report. Ethical clearance for all procedures was obtained from the NHS National Research Ethics Service (Ref. 11/NW/0382). All participants provided written informed consent. This study has been preregistered at https://osf.io/83zm7/. After documenting the preregistration, we reworded the subgroups ‘moderate’ and ‘severe’ recurrent Major Depressive Disorder (rMDD) into moderate recurrence severity and high recurrence severity to clarify that the subgroups document a staging and progression of the severity of recurrence, and not explicitly severity of the symptoms. Data retrieval from the Biobank occurred in October 2023.

Mental health questionnaire data were gathered at the time of imaging. Exclusion criteria for participants were: having had a substance or behavior addiction, a neurodegenerative or stroke-related disease, a prescription of antipsychotics, or manic or bipolar symptoms following Smith *et al.* guidelines (D. J. Smith, Nicholl, et al., 2013). To limit old age-introduced variability, participants attending the first imaging phase at age 70 years or over were excluded. Additionally, we excluded participants who withdrew consent or with missing data for any of the features used.

The focus of our study was on the amygdala’s responses to facial stimuli compared to geometric shapes, as assessed using a shortened version of the Hariri task (Hariri, Tessitore, Mattay, Fera, & Weinberger, 2002). During the fMRI scan, participants were asked to match the representation at the top with either the left or right representation at the bottom. This task alternated between experimental blocks featuring (negative) facial stimuli and control blocks involving shapes. We evaluated both the BOLD median (field ID 25052) and BOLD 90th percentile (field ID 25767) (faces–shapes) contrasts separately. Detailed information on the processing of imaging data can be accessed at https://biobank.ctsu.ox.ac.uk/crystal/refer.cgi?id=1977.

### NM framework

For NM, based on previous NM studies on mental disorders, covariates used for both the BOLD median and BOLD 90th percentile were age, sex, and head motion during the scan (Rutherford et al., 2023). We split the HC sample into 80% training data for charting the normative trends using NM and 20% testing data for significance assessment. This split was done by first taking all the HCs that attended both imaging visits (8%) into the test set, to retain as much longitudinal data as possible for analysis. The other 12% was sampled randomly from the remaining HC participants. We used a Bayesian generalized linear model as the NM, which is scalable to large datasets, while retaining relatively fast modeling time. The model was fitted and applied using the stan_glm function from the rstanarm package in R (Goodrich, Gabry, Ali, & Brilleman, 2020). The default normally distributed prior was used, with four chains of 2,000 iterations (1,000 warmup) for fitting the Bayesian model. Using the Bayesian Information Criterion (BIC) score, a linear model was compared to a cubic spline model, by measuring goodness-of-fit, penalized by the degrees of freedom of the model.

### Classification

We categorized the frequency of lifetime recurrent MDEs (rMDD) as recurrence severity into three groups (separate from HCs). Inclusion criteria into this staging model are largely based on Smith et al., having had at least one episode of feeling depressed or anhedonic for at least 2 weeks (field ID 4609,5375), and having visited either a doctor or psychiatrist for nerves, anxiety, or depression (field ID 2090,2100). We identified single episodes, moderate recurrence severity (two to five episodes), and high recurrence severity (≥6 episodes) among participants. These criteria resulted in a prevalence closely resembling Smith et al.’s findings (Supplemental Figure S1 and Table S2). In the UKB, the RDS-4 (henceforth referred to as RDS) questionnaire was used to determine the current existence of depressive symptoms, concerning symptoms over the past 2 weeks before the imaging visit. The questionnaire consisted of four questions about typical symptoms and their frequency, given a scale ranging from 1 (*not at all*) to 4 (*nearly every day*). The four question scores are then added to form a total score ranging from 4 to 16. The presence of current depressive symptoms was defined as RDS > 8, concurrent with moderate severity of depression based on PHQ ≥ 10 (Dutt et al., 2022; Manea, Gilbody, & McMillan, 2012). Therefore, remission was defined as RDS ≤ 8. Current and ongoing AD use of participants was captured through a verbal interview within assessment centers from which AD medication codes are recorded at the time of scanning (see Supplemental S3). The metric on AD use covers current prescription medication that is being taken by the participant at the time of scanning, as additionally confirmed during a verbal interview at the assessment center. When excluding AD use in the longitudinal analysis, we exclude participants who are taking AD medication at either time point of scanning.

### Statistical analysis

We used identifiers (i.e. 1a.1) for each analysis for reading clarity and added an overview of the data being used and variable of interest in Supplemental Table S4. To adhere to the assumptions of a parametric analysis of variance (ANOVA), ordered quantile normalization was applied to both NM scores and unaltered BOLD features (Peterson & Cavanaugh, 2020). We performed nine baseline cross-sectional analyses, split into five analyses focused on trait effects (nondepressed; RDS ≤ 8) and four incorporating (presumable) symptomatic MDE (RDS > 8). As cross-sectional analysis (1), we performed Fisher’s one-way ANOVA between HCs, single episode, moderate, and severe recurrence severity for subjects currently in remission (RDS ≤ 8). This was done unaltered and through the NM model converted into deviation scores (analysis 1a.1), for both the BOLD median and 90th percentile features. Based on the results thereof, we chose features for further analysis. As exploratory analyses (not documented in the preregistration), the effect of body mass index (BMI), education, and employment as covariates in the NM framework was assessed within our analysis. We additionally tested covariates/confounders and their deviations between groups, such as BMI, education, and employment, to verify their effect on results.

As previously mentioned, the HC group analyzed was specifically tailored to incorporate as much repeat imaging data as available, to retain more longitudinal data for statistical analysis. Therefore, the ratio of participants who attended a second imaging visit is much higher in the HC group than in the depression group. We performed a sensitivity analysis to cover any potential bias introduced due to this imbalance, by randomly removing participants within the HC group with repeat imaging data available, such that the ratio is the same for both groups, and repeated analysis 1a.1 with this subgroup.

Moderate and high recurrence severity were pooled in a repeat analysis (analysis 1a.2) to gain statistical power. To assess the effect of ADs, analyses 1a.1 and 1a.2 were repeated for participants without AD use (analyses 1a.3 and 1a.4, respectively). Finally, the interaction with AD medication was analyzed through a two-way (recurrence-severity stage; single episode/moderate/severe by AD medication; yes/no) ANOVA interaction model (analysis 1a.5).

State analyses (analyses 1b) were centered around the effects of symptomatic MDEs (RDS > 8). We repeated analysis 1a.1 for the group with symptomatic MDE (analysis 1b.1). Subsequently, we tested for a possible interaction between current depressive symptoms and recurrence severity, through a two-way ANOVA interaction model (analysis 1b.2). An independent *t*-test was performed, comparing a subset of the HC group containing no current depressive symptoms (RDS = 4) to moderate and higher symptoms (RDS > 8, analysis 1b.3), which we repeated excluding participants using AD medication (1b.4).

For our longitudinal analysis (2), we performed three separate ANOVA analyses per lifetime recurrence-severity group (analysis 2a.1–3), analyzing the interaction between time (imaging visits at baseline and at 2 years) and an increase in MDEs over time. The increase in MDEs was derived from comparing the recalled MDEs by the participant at both imaging visits. To have a sufficiently homogeneous sample, we focused on the group in remission during both scans. To increase sample size in the longitudinal analysis, moderate and high recurrence-severity groups were combined to represent a total of 107 participants experiencing 2+ recurrent episodes at the first imaging visit (T0). Increase in episodes was categorized into no additional episodes, one additional episode, and more than one additional episode in between phases. Finally, we repeated these longitudinal analyses (in participants with remitted MDD), while excluding AD medication (analysis 2b.1–2).

For all the above frequentist analyses, we reported significance when *p* < 0.05, with the effect size in terms of partial omega-squared (*ω* ^2^). Post-hoc paired *t*-tests were performed, correcting for multiple comparisons through Holm-adjusted *p*-values. To quantify the probability in favor of the proposed hypothesis as opposed to the null hypothesis, a Bayesian ANOVA was then conducted and its Bayes factor (BF) reported, in order.

## Results

### Demographics

Table 1 shows sample sizes and their respective demographics and clinical characteristics per defined group (six groups: HCs [divided into NM estimation and analysis samples], single depressive episode, moderate recurrence severity, high recurrence severity, and symptomatic MDE [RDS > 8]). Statistical analysis in Table 1 was not part of the preregistration. Compared to the HC analysis set, age, gender, employment, education, and BMI were identified as significantly deviating covariates in depressive (history) groups relative to HCs, of which age and gender were included in the NMs. Ethnicity between groups deviated significantly in the moderate recurrence severity and symptomatic MDE groups for Asian and mixed backgrounds, respectively.

**Table 1.** Demographics and clinical characteristics of each relevant subgroup (total *N* = 11,334)

	HC1. Healthy controls (normative modeling group) (*N* = 6,442)			HC2. Healthy controls (Analysis group) (*N* = 1,610)			1. Single depressive episode (*N* = 926)		
Characteristic	N	%		N	%	*p* (Fisher’s exact test vs. HC1)	N	%	*p* (Fisher’s exact test vs. HC2)
Female	3332	51.7		796	49.4	0.11	584	63	**<0.001**
Race/ethnicity									
Black	46	0.7		8	0.5	0.40	6	0.6	0.59
Asian	102	1.6		21	1.3	0.50	10	1.1	0.71
Mixed	37	0.6		8	0.5	0.85	4	0.4	1.00
Other/prefer not to answer	49	0.8		10	0.6	0.63	6	0.6	1.00
White	6206	96.4		1561	97.1	0.26	900	97.2	0.81
Employed	3028	47		830	51.6	**0.001**	480	51.8	0.90
College/University degree	3335	51.8		839	52.1	0.82	501	54.1	0.34
Antidepressant use	–	–	–	–	–	–	49	5.3	–

	Mean	SD		Mean	SD	*p* (*t*-test vs. HC1)	Mean	SD	*p* (*t*-test vs. HC2)
Age (years)	61.2	5.8		60.2	5.9	**<0.001**	60.2	5.7	0.95
BMI	25.5	5.8		25	6.5	**0.002**	25.8	6.7	**0.002**

	2. Moderate recurrence-severity (2–5 episodes) (*N* = 1,530)			3. High recurrence severity (6+ episodes) (*N* = 549)			4. Symptomatic MDE (RDS > 8) (*N* = 277)		
Characteristic	*N*	%	*p* (Fisher’s exact test vs. HC2)	*N*	%	*p* (Fisher’s exact test vs. HC2)	*N*	%	*p* (Fisher’s exact test vs. HC2)
Female	1093	71.4	**<0.001**	344	62.7	**<0.001**	187	67.5	**<0.001**
Race/ethnicity									
Black	8	0.5	1.00	4	0.7	0.51	0	0	0.61
Asian	8	0.5	**0.02**	2	0.4	0.09	4	1.4	0.78
Mixed	6	0.4	0.79	2	0.4	1.00	6	2.2	**0.01**
Other/prefer not to answer	11	0.7	0.83	5	0.9	0.55	0	0	0.37
White	1497	97.8	0.15	536	97.6	0.46	267	96.4	0.58
Employed	848	55.4	**0.03**	287	52.2	0.80	174	62.8	**<0.001**
College/University degree	856	55.9	**0.03**	305	55.6	0.17	120	43.3	**0.01**
Antidepressant use	235	15.4	–	119	21.7	–	95	34.3	–

	Mean	SD	*p* (*t*-test vs. HC2)	Mean	SD	*p* (*t*-test vs. HC2)	Mean	SD	*p* (*t*-test vs. HC2)
Age (years)	59.6	5.7	**0.004**	60	5.8	0.4	57.9	5.6	**<0.001**
BMI	26	6.3	**<0.001**	26.3	6.6	**<0.001**	27.8	7.8	**<0.001**

HC2 (analyzed group) reported *p*-values are with respect to HC1 (HC used for fitting the NM), depression-related groups are compared to HC2. Significant differences (*p* < 0.05) are highlighted in bold. MDE, major depressive episodes; RDS, recent depressive symptoms.

### Cross-sectional state analysis

For NM, BIC scores revealed a linear Bayesian model as a better (penalized) fit than adding cubic splines (Supplemental Figure S5–S8 and Table S9). Therefore, this model was adopted for further analysis.

For the main trait analysis (analysis 1a.1), a statistically significant association between BOLD median response and recurrence of depression was found in the baseline ANOVA analysis within recurrence severity, in the feature scores processed by NM (*F* = 3.06, df = 3, *p* < 0.01, *ω* ^2^ = 0.002, Figure 1). Post-hoc examinations of the deviation scores extracted through applying NM displayed a significant Holm-adjusted pairwise difference between the HC and high recurrence-severity group (*p*_Holm_ = 0.02, Figure 1), whereas Bayesian ANOVA analysis provided moderate support for the null hypothesis (BF_10_ = 0.15). Repeating the same Holm-adjusted *t*-test with the unaltered BOLD median feature did not show a significant difference (*F* = 1.59, df = 3, *p* = 0.19). A visual comparison of the distributions and the effect of NM can be found in Supplemental Figures S10 and S11.

To properly account for deviations in the BMI and explore sensitivity, we repeated the analysis, adding BMI to the NM as a covariate, which resulted in similar results (*F* = 3.67, df = 3, *p* = 0.01, *ω* ^2^ = 0.002) and post-hoc comparison between HC and high recurrence-severity (*p*_Holm_ = 0.02). Similarly, adding current employment (*F* = 3.42, df = 3, *p* = 0.02, *ω* ^2^ = 0.002, *p*_Holm_ = 0.02) and achieving a college or university degree (*F* = 3.02, df = 3, *p* = 0.03, *ω* ^2^ = 0.001, *p*_Holm_ = 0.03) as binary covariates to the NM did not change results.

We performed a sensitivity analysis to quantify any potential bias introduced due to the imbalance of repeat visits per subgroup, by randomly removing 951 out of the 1,037 within the HC group with repeat imaging data available, such that the ratio is the same for both groups (13%). We then repeated analysis 1a.1 with this subgroup and found no deviating results (*F* = 2.70, df = 3, *p* = 0.04, *ω* ^2^ = 0.001, *p*_Holm_ = 0.04, Supplemental Figure S12). Additionally, we analyzed differences in RDS scores and ratio of repeat-imaging visits between depression-related groups (Supplemental Table S13). We found no significant differences between groups for repeat imaging visit ratios (*F* = 1.59, df = 3, *p* = 0.19) but found a significant difference in RDS scores between the three remitted groups (single episode, moderate, and high recurrence severity) at T0 (*F* = 88.2, df = 2, *p* < 0.01, *ω* ^2^ = 0.05), as well as T1 (*F* = 7.42, df = 2, *p* < 0.01, *ω* ^2^ = 0.03). Additionally, for the HC and high recurrence-severity groups, we found a significant difference in the RDS score between the phases T0 and T1, despite the thresholding of these groups at RDS ≤ 8. See Supplemental Table S13 for further details on these analyses.

The BOLD 90th percentile did not yield significant findings when repeating analysis 1a.1 with this feature, for both unaltered feature values (*F* = 1.07, df = 3, *p* = 0.36) and NM-adjusted deviation scores (*F* = 0.16, df = 3, *p* = 0.92) (Supplemental Figure S14). Further analyses (analysis 1a.2 and beyond) were performed using the NM-adjusted BOLD median feature, and the NM was confined to the original setup with covariates age, gender, and head motion during the scan as described in previous work (Rutherford et al., 2023).

### Additional recurrence-severity (trait) analysis

**Figure 2.**
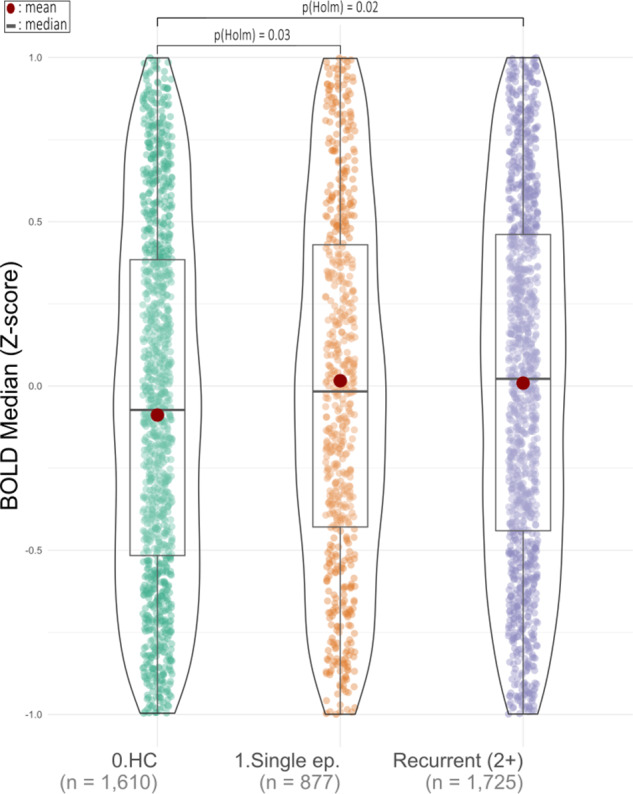
Analysis of participants not on antidepressant medication with a pooled recurrence (2+) group, combining moderate and high recurrence severity (analysis 1a.4). The BOLD median Z-score *y*-axis is clipped at [−1, 1] for clarity. The top bar shows significant post-hoc pairwise *t*-tests. HC, healthy control. Single ep., single episode.

Combining moderate and high recurrence severity (analysis 1a.2) into a single group resulted in a further decrease in Holm-adjusted pairwise comparison to below 0.01 between the HC- and the 2+ episodes recurrence-severity group. However, effect size remained similar (analysis 1a.2, *F* = 5.35, df = 2, *p* < 0.01, *ω* ^2^ = 0.002, *p*_pairwise-Holm_ < 0.01, Supplemental Figure S15). For analysis 1a.3, where we exclude AD-medicated participants, we excluded 49 (5.3%) in the single-episode group, 235 in the moderate (15.4%), and 119 in the high (21.7%) recurrence-severity group. This did not change the results compared to analysis 1a.1 (*F* = 2.91, df = 3, *p* = 0.03, *ω* ^2^ = 0.002, *p*_pairwise-Holm_ = 0.02, Supplemental Figure S16). After merging the moderate and high recurrence-severity groups (2+ episodes) and excluding AD-medicated participants (analysis 1a.4), Holm-adjusted pairwise comparisons indicated an additional significant deviation between the HC group and both classes of depression recurrence-severity history (analysis 1a.4, *F* = 4.78, df = 2, *p* < 0.01, *ω* ^2^ = 0.002, *p*_pairwise-Holm_ = 0.03/0.02, Figure 2). We found no significant recurrence severity by AD use interaction (analysis 1a.5, *F* = 2.8, df = 2, *p* = 0.06, *ω* ^2^ = 0.001, Supplemental Figure S17).

### Symptomatic MDE (state) analysis

Within the symptomatic MDE group, who were depressed when scanned (RDS > 8, analyses 1b), no statistically significant division between recurrence-severity stages was found (1b.1, *F* = 0.47, df = 2, *p* = 0.62, Supplemental Figure S18). Furthermore, we found no significant recurrence severity by state (depressed or remission) interaction (analysis 1b.2, *F* = 0.33, df = 2, *p* = 0.72, Supplemental Figure S19). Two separate *t*-tests were conducted to analyze state and AD medication effects. For these analyses, the HC were limited to RDS-4 = 4 (no current depressive symptoms over the last 2 weeks). A visual overview of the comparison of these groups can be found in Supplemental Figure S20. No significant differences were found between the (RDS-4 = 4 limited) HC and the symptomatic MDE group (RDS-4 > 8) (analysis 1b.3, *t* = −1.24, df = 1,193, *p* = 0.21), with the same result when AD-medicated participants were removed for the current onset group (analysis 1b.4, *t* = −0.05, df = 1,098, *p* = 0.96).

### Longitudinal analysis

**Figure 3.**
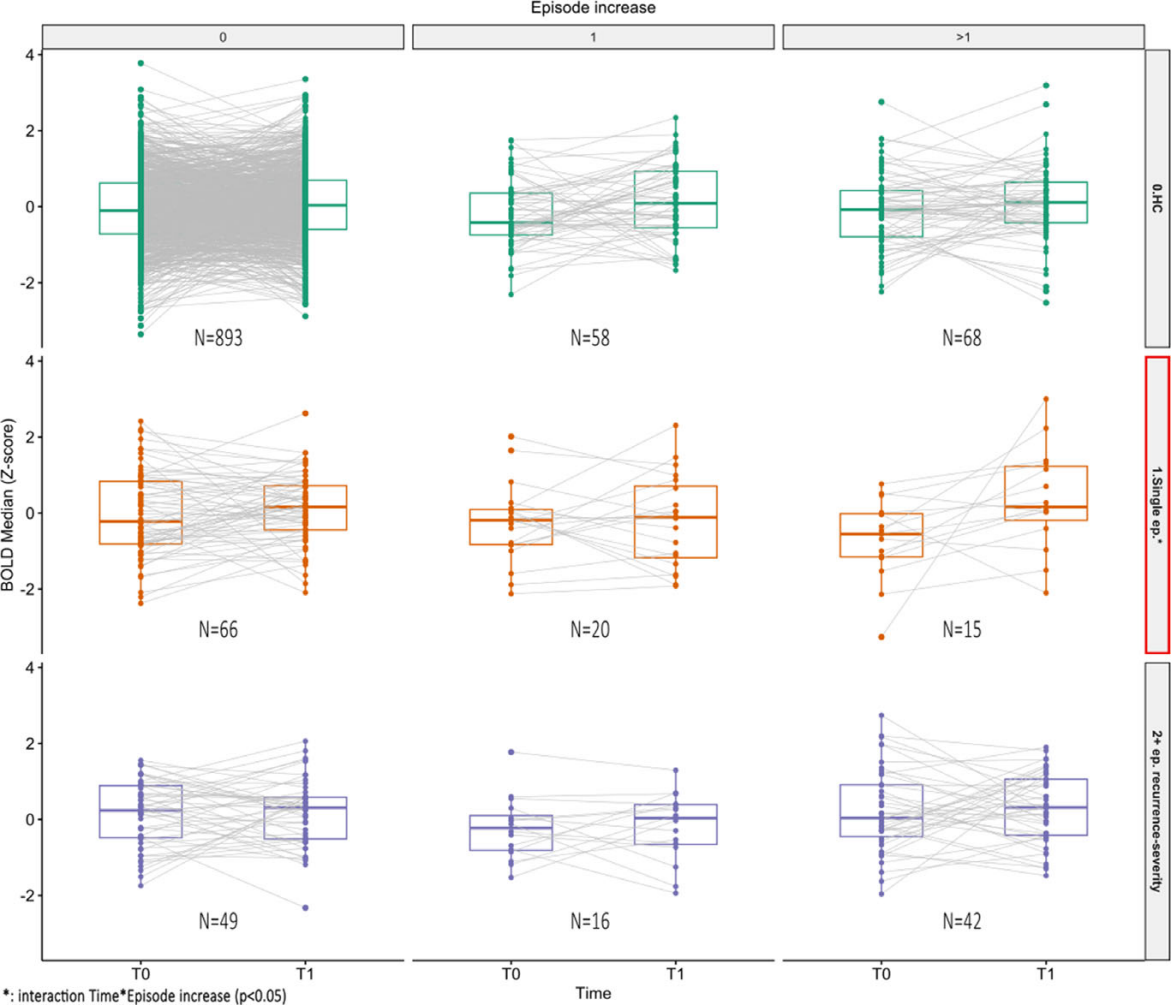
Longitudinal data of the (NM-adjusted) amygdala BOLD signal (analysis 2a.1–3), with episode increase between repeated visits T0 and T1 in columns (0, 1, and > 1) and lifetime recurrence classification at the first visit in rows. Moderate and high recurrence severity are combined to increase power. A significant interaction between time and increase in episodes was found in the highlighted (*) single-episode group (analysis 2a.2).

Our longitudinal analyses (analysis 2) separately analyzed 3 groups, consisting of 1,019 HCs, 101 single episode, and 107 (combined moderate + high) recurrence-severity (remitted) participants, respectively, identified during the first imaging visit (T0). Figure 3 shows the change over time in mean NM-adjusted amygdala signal grouped by the initial lifetime recurrence classification and the relative increase in episodes. The HC group (analysis 2a.1, *F* = 0.86, df = 2, *p* = 0.42) and recurrence-severity group (analysis 2a.3, *F* = 0.02, df = 2, *p* = 0.98) did not yield significant interactions. Within the group with a single episode at baseline, a significant time by episode-increase interaction was found (analysis 2a.2, *F* = 3.48, df = 2, *p* = 0.03, *ω* ^2^ = 0.017). Post-hoc analysis indicated a significant increase between T0 and T1 within the group experiencing more than one episode between imaging visits (*p*_pairwise-Holm_ = 0.03, Table 2). A Bayesian ANOVA analysis demonstrated weak evidence for the null hypothesis of equality (BF_10_ = 0.33). We then repeated the longitudinal analyses, excluding 26 (13% of the initial groups for longitudinal analyses) remitted MDD patients with AD medication at either time point (T0 or T1; Supplemental Table S21; analysis 2b.1–2). We then found no significant interactions within the single episode (analysis 2b.1, *F* = 2.81, df = 2, *p* = 0.06) or recurrent-severity groups (analysis 2b.2, *F* = 0.03, df = 2, *p* = 0.97) (Supplemental Figure S22).

**Table 2.** Post-hoc longitudinal analysis of the single depressive episode group (measured at T0) (analysis 2a.2), showing the interaction of imaging visit (T0: first imaging visit, T1: repeat imaging visit) and episode increase (categorized into 0, 1, and >1)

Post-hoc longitudinal analysis – Single episode (at T0) group					
Group 1	Group 2	Mean difference (Group 1 − Group 2) of NM-adjusted amygdala BOLD signal	SE	*t*	*p*_holm_
0, T0	1, T0	0.22	0.27	0.81	1
	>1, T0	0.66	0.31	2.17	0.41
	0, T1	−0.15	0.16	−0.95	1
	1, T1	0.16	0.27	0.61	1
	>1, T1	−0.40	0.31	−1.30	1
1, T0	>1, T0	0.44	0.37	1.21	1
	0, T1	−0.37	0.27	−1.35	1
	1, T1	−0.05	0.29	−0.19	1
	>1, T1	−0.62	0.37	−1.69	1
>1, T0	0, T1	−0.81	0.31	−2.66	0.12
	1, T1	−0.50	0.37	−1.36	1
	**>1, T1**	**−1.06**	**0.33**	**−3.21**	**0.03**
0, T1	1, T1	0.32	0.27	1.16	1
	>1, T1	−0.25	0.31	−0.81	1
1, T1	>1, T1	−0.56	0.37	−1.55	1

Significant difference (*p* < 0.05) is highlighted in bold. The *p*-value is Holm-adjusted for 15 comparisons.

## Discussion

With our analyses of NM-adjusted amygdala BOLD signal in the UKB, we convey that amygdala responses to negative faces are not a biomarker for the presence of a current MDE (state) in the general population of the UKB. However, we demonstrate that this amygdala BOLD signal, rather, is a trait representative of an individual’s history of recurrent depressive episodes. This is supported by the significant effect of single or recurrent episodes versus HCs when excluding AD use in remitted patients and by our longitudinal analyses. By a reduction of amygdala responses, AD use appeared to confound the effects of recurrence. Because clinical evidence shows that an increased number of episodes is associated with increased recurrence risk (Figueroa et al., 2015), the association of the amygdala response with recurrent depressive episodes suggests a possible association with increased vulnerability for MDEs and, as such, may also be indicative of an individual’s risk of recurrence (although not studied here).

### Cross-sectional analysis

Our (normative) cross-sectional results extend previous research by Tamm et al., showing an absence of amygdala response in association with symptom severity. However, we add to this finding by showing an association of amygdala response with recurrent MDEs (Tamm et al., 2022). Combining the history of previous MDEs, symptomatic MDE, and AD medication, applying NM in both stratified and interaction analyses proved vital to understanding their complex interplay, as shown.

NM was key in identifying effects between groups at increasing stages of recurrence-severity symptoms, by expressing findings as deviations from expected norms and, thus, better accommodating the variability in group-specific characteristics at a trait level. These findings underscore the potential of NM approaches to elucidate subtle but critical differences that may inform targeted interventions or personalized applications.

### Longitudinal analysis

In our longitudinal analysis, the group in remission of a single MDE showed a significant increase in amygdala response when experiencing >1 new MDE within 2 years of follow-up. Of note, this effect was observed while being in remission of the MDEs at both time points. This result substantiates the Kindling theory (Post, 1992), suggesting that each relapse of depression leaves a ‘scar’. We hypothesize that this could be relevant in the amygdala, becoming more sensitive (expressed by heightened activity) to negative information. Absence of this time by an increase in episode interaction effect in the moderate/severe rMDD group could be explained by a ceiling effect for the amygdala BOLD signal occurring after numerous recurrences. The lack of interaction of time and occurrence of depressive episodes in the HC group might be explained by a difference in the assessment of a depressive episode between the HC group experiencing new depressive episode(s) and the group who already had a ‘single episode’ at baseline. In the latter group, the depressive episode was additionally validated by requiring a visit to a professional for nerves, anxiety, or depression, which was not necessary for the HC to claim a new depressive episode. Therefore, this apparent difference in change due to an incident MDE might be biased by ascertainment differences. If so, this effect might then also be considered as a validation of the necessity of requiring a healthcare professional visit in conjunction with self-reported depressive symptoms in population samples, as proposed by Smith, Nicholl, et al. (2013).

### AD medication

Unexpectedly, our analyses showed that AD medication did not result in a significant decrease in amygdala responses in the current symptom group in the longitudinal analysis, despite being in remission. With the removal of 13% of the initial sample using ADs, we might have reduced power to find results. However, we suspect that, alternatively, the exclusion of AD-using participants might have caused a selection bias because increased recurrence severity (and thus susceptibility for new episodes) will be associated with a higher prevalence of AD use. This aligns with the higher prevalence of AD medication found in the recurrence-severity groups (from 5.3% in the single episode to 21.6% in the high recurrence severity). Additionally, it is important to note that in our longitudinal analysis, AD medication was not equal at both time points for all participants. Hence, the removal of AD-medicated participants in the longitudinal analysis will have had variable influences in each subgroup.

### Limitations

Despite the strength of our study, which analyzed the largest sample size available and combined functional neuroimaging with depressive symptoms, both longitudinally and cross-sectionally, several limitations should be mentioned. First, our stratification split up the large dataset (e.g. the single episode (sub)group broke up into smaller episode-increase subgroups with small sample sizes [up to *n* = 15]). Second, previous studies have reported that depression groups with and without early-life trauma, as well as comorbid anxiety, may differ in their emotional processing – which is also reflected in amygdala activation (Grant, Cannistraci, Hollon, Gore, & Shelton, 2011). The UKB measure indicating ‘having visited either a doctor or psychiatrist for nerves/anxiety/depression’ combines these three indications and, therefore, we cannot strictly isolate anxiety from the reported purpose of seeking professional help. Third, due to the recurrence of episodes being collected retrospectively, inaccuracy and bias might arise when recalling the lifetime depressive episode count for the participants. The small effect sizes found may be a product of this inaccuracy, in addition to increased variability inevitably found in population studies. Nevertheless, this suggests limited clinical applicability per se, but corroborates the existence of a kindling phenomenon. Additionally, NM proved to be an important factor for picking up on the nuanced effects of the amygdala BOLD contrasts, as without NM, we failed in doing so. Further analysis within the symptomatic MDE sample could be of interest due to the relatively limited sample size in this study (*N* = 277). This limitation is, however, expected in a volunteer-based population study. Similarly, this underrepresented sample hindered analysis of interactions between symptomatic MDE and lifetime recurrence severity, where we found no effect. Once larger sample sizes become available for the high-RDS group (>8) longitudinally, future studies may analyze further interactions between state and trait variables and the amygdala signal.

Lastly, reproducibility of fMRI-derived neuronal responses in mental health studies is of major concern (Flournoy et al., 2024). However, relevant for this study, it was recently suggested that neural responses to emotional cues may be more reflective of intraindividual variation over time rather than measurement errors. Although test–retest reliability was low within-person, within-session internal consistency of the BOLD signal was higher, and within-person fluctuations across sessions explained almost half the variance in voxel-level neural responses (Flournoy et al., 2024). As a result, single-participant prediction for MDD using fMRI remains challenging, as found by recent reviews on both AD treatment response (Gerlach et al., 2022) and classification (Winter et al., 2022). Finally, it is important to acknowledge the ongoing challenges in processing fMRI data and the need for further advancements to ensure reproducibility and reliability of results in fMRI tasks (Flournoy et al., 2024; Specht, 2020).

In conclusion, based on the huge sample in the UKB, our study provides better insights into the relationship between amygdala BOLD-response, the number of depressive episodes and relapses/recurrences in MDD, relative to previous analyses (Tamm et al., 2022). For the middle-aged and elderly participants (up to age 70 years) included in this study, the amygdala response is associated with the number of recurrent episodes in (remitted) depression. Our longitudinal analysis is supportive of a ‘kindling’ effect of additional MDEs on the amygdala responsivity to negative information, measurable during remission. This offers new insight into the kindling effect of depression on the brain and how amygdala responses might be helpful as a marker for MDD recurrence severity.

## Supporting information

Van Den Berg et al. supplementary material Van Den Berg et al. supplementary material

## Supplementary material

The supplementary material for this article can be found at http://doi.org/10.1017/S0033291725101797.
