# Supplementary material for: Normative amygdala fMRI response during emotional processing as a trait of depressive symptoms in the UK Biobank
Source: Psychol Med. 2025 Oct 8;55:e304. doi: 10.1017/S0033291725101797 (PMC12527494; doi:10.1017/S0033291725101797)

**Supplementals**

This file contains figures, tables and text supplementary to the manuscript *‘Normative Amygdala fMRI Response during Emotional Processing as a Trait of Depressive Symptoms in the UK Biobank’*. Numbering is continuous throughout for easier referencing.

**Figure S1: Classification of recurrent MDD according to Smith et al., updated for the Kindling theory (stratifying the amount of lifetime episodes)**


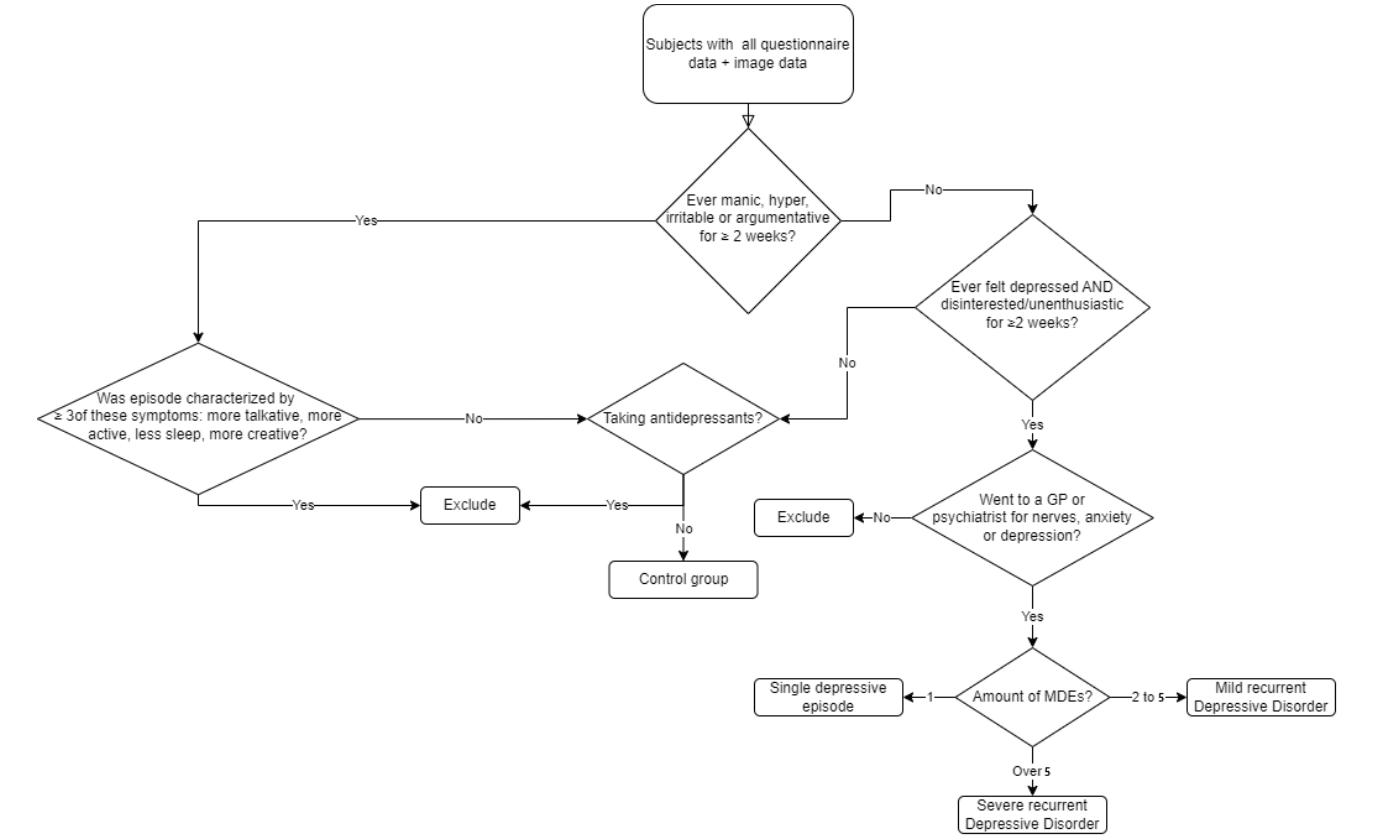


**Table S2: Overlap in classification by Smith et al. and our updated classification of lifetime MDD recurrence**

|  |  | **Updated (kindling) definition** | |  |
| --- | --- | --- | --- | --- |
|  |  | **2-5 episodes, seen GP OR Psych.** | **6+ episodes, seen GP OR Psych.** | **Total (% of pop.)** |
| **Smith et al. Definition** | **2+ episodes, seen GP** | 1262 | 424 | 1686 (15.1%) |
|  | **2+ episodes, seen Psych.** | 380 | 269 | 649 (5.8%) |
|  | **Total (% of pop.)** | 1642 (14.7%) | 693 (6.2%) |  |

**S3: Medication and illness codes used in the UK Biobank for antidepressants, antipsychotics, neurodegenerative- and stroke related illnesses**

Treatment/medication codes for antidepressants (Field 20003):

allegron 10mg tablet 1140867820

amitriptyline hydrochloride+perphenazine 10mg/2mg tablet 1140867948

amitriptyline 1140879616

amitriptyline+chlordiazepoxide 12.5mg/5mg capsule 1140867938

amoxapine 1140867774

anafranil 10mg capsule 1140867690

Atomoxetine 1141199446

Bupropion 1141176854

cipralex 5mg tablet 1141190158

cipramil 10mg tablet 1141151946

citalopram 1140921600

clomipramine 1140879620

cymbalta 30mg gastro-resistant capsule 1141201834

depixol 3mg tablet 1140867152

desipramine 1140879624

dosulepin 1140909806

dothiepin 1140879628

doxepin 1140867640

duloxetine 1141200564

Dutonin 1140917466

edronax 4mg tablet 1141151982

efexor 37.5mg tablet 1140916288

Elavil 1140867658

Eldepryl 1140872348

escitalopram 1141180212

faverin 50mg tablet 1140867860

fluanxol 500micrograms tablet 1140867952

fluoxetine 1140879540

flupenthixol 1140867150

flupentixol 1140909800

fluphenazine hydrochloride+nortriptyline 1.5mg/30mg tablet 1140867940

fluvoxamine 1140879544

Gamanil 1140882310

imipramine 1140879630

isocarboxazid 1140867856/1140910504

lofepramine 1140867726

Lomont 1141146062

Ludiomil 1140867784

lustral 50mg tablet 1140867884

manerix 150mg tablet 1140867922

maoi - tranylcypromine 1140910820

manerix 1140867922

maprotiline 1140879552

Marplan 1140867858

mianserin 1140879556

mirtazapine 1141152732

moclobemide 1140867920

molipaxin 50mg capsule 1140882244

nardil 15mg tablet 1140867852

Nefazodone 1140917460

nortriptyline 1140867818

oxactin 20mg capsule 1141174756

parnate 10mg tablet 1140867916

paroxetine 1140867888

phenelzine 1140867850/1140910704

prothiaden 25mg capsule 1140867624

protriptyline 1140879632

prozac 20mg capsule 1140867876

reboxetine 1141151978

selegiline 1140879668

seroxat 20mg tablet 1140882236

sertraline 1140867878

st john's wort/hypericum [ctsu] 1201

sinequan 10mg capsule 1140882312

Strattera 1141199460

surmontil 10mg tablet 1140867758

tofranil 10mg tablet 1140867712

tranylcypromine 1140867914

tranylcypromine+trifluoperazine 10mg/1mg tablet 1140867944

trazodone 1140879634

trimipramine 1140867756

triptafen tablet 1140867934

Tryptizol 1140867668

tryptophan product 1140867960

venlafaxine 1140916282

Viloxazine 1140879688

Vivalan 1140867770

yentreve 20mg gastro-resistant capsule 1141200570

Zelapar 1141169666

zispin 30mg tablet 1141152736

Participants with a history of antipsychotics are excluded, defined by the following medications:

Abilify 5mg tablet 1141202024

Amisulpride 1141153490

Aripiprazole 1141195974

Benperidol 1140867078

Camcolit 250 tablet 1140867494

Carbagen SR 200mg M/R tablet 1141171566

Carbamazepine 2038459704

Carbamazepine product 1140872064

Chlorpromazine 1140879658

Clopixol 2mg tablet 1140867342

Clozapine 1140867420

Clozaril 25mg tablet 1140882320

Convulex 150mg E/C capsule 1140872216

CPZ-Chlorpromazine 1140910358

Denzapine 25mg tablet 1141200458

Depakote 250mg E/C tablet 1141172838

Dolmatil 200mg tablet 1140867306

Dozic 1mg/ml oral liquid 1140867180

Epilim 100mg crushable tablet 1140872200

Fentazin 2mg tablet1140867210

Fluphenazine decanoate 1140867398

Fluphenazine 1140882098

Haldol 5mg tablet 1140867184

Haloperidol 1140867168

Largactil 10mg tablet 1140863416

Levomepromazine 1140909802

Liskonum 450mg M/R tablet 1140867498

Lithium product 1140867490

Lithonate 400mg M/R tablet 1140910976

Methotrimeprazine 1140867118

Modecate 12.5mg/0.5ml oily injection 1140867456

Olanzapine 1140928916

Orlept 200mg E/C tablet 1140872268

Pericyazine 1140867134

Perphenazine 1140867208

Pimozide 1140867218

Piportil depot 50mg/1ml oily injection 1140867572

Pipothiazine 1140879674

Pipotiazine 1140909804

Priadel 200mg M/R tablet 1140867504

Prochlorperazine 1140868170

Promazine 1140879746

Quetiapine 1141152848

Risperdal 0.5mg tablet 1141177762

Risperidone 1140867444

Serenace 500micrograms capsule 1140867092

Seroquel 25mg tablet 1141152860

Sodium valproate 1140872198

Stelazine 1mg tablet 1140867244

Stemetil 5mg tablet 1140868172

Sulpiride 1140867304

Tegretol 100mg tablet 1140872072

Thioridazine 1140879750

Trifluoperazine 1140868120

Valproic acid 1140872214

Zaponex 25mg tablet 1141201792

Zuclopenthixol 1140882100

Zyprexa 2.5mg tablet 1141167976

Medical history under ICD10 diagnosis is available under feature 41270 and illnesses are also self-reported under feature 20002.

Having an ICD10 code present starting with I6 (Cerebrovascular diseases) flags participants as having a history of a stroke-related disease, as well as the following codes for self-reported illness:

1081 stroke

1082 transient ischaemic attack

1083 subdural haemorrhage /haematoma

1086 subarachnoid haemorrhage

1425 cerebral aneurysm

1491 brain haemorrhage

1583 ischaemic stroke

Having an ICD10 code present starting with G3 flags participants as having a neurodegenerative-related disease, aswell as the following codes for selfreported illness:

1258 chronic/degenerative neurological problem

1259 motor neurone disease

1260 myasthenia gravis

1261 multiple sclerosis

1262 parkinsons disease

1263 dementia/alzheimers/cognitive impairment

**Table S4: Analysis identifier overview, specifying subgroup and variables of interest**

| **Analysis group** | **Identifier** | **Description** |
| --- | --- | --- |
| **Recurrence-Severity Analysis** (remitted; restricted to RDS≤8)  **Imaging visit:** T0 | 1a.1 | **One-way ANOVA:**   - Groups:   - HC, Single episode, moderate recurrence-severity, high recurrence-severity   **Features:**   - Repeated for each feature separately (4x):   - BOLD-Median (NM/unaltered)   - BOLD-90th (NM/unaltered)   **NM-derived BOLD-Median is used for further analyses** (no significant effect in BOLD 90th or unadjusted feature values) |
|  | 1a.2 | Repeat of analysis 1a.1 with **One-way ANOVA**:  Moderate+High recurrence groups combined |
|  | 1a.3 | Repeat of analysis 1a.1 with **One-way ANOVA**: Non-AD medicated participants (AD participants removed) |
|  | 1a.4 | Repeat of analysis 1a.1 with **One-way ANOVA**: Moderate+High groups combined AND Non-AD medicated participants |
|  | 1a.5 | Repeat of analysis 1a.1 with **Two-way ANOVA:** Interaction recurrence severity (Single episode, moderate recurrence-severity, high recurrence-severity) by AD medication (yes/no) |
| **Current onset analysis** (Depressive symptoms at time of scanning; RDS>8)  **Imaging visit:** T0 | 1b.1 | **One-way ANOVA**: Recurrence-severity (single episode, moderate recurrence-severity, high recurrence-severity) categorized within current onset (RDS>8) |
|  | 1b.2 | **Two-way ANOVA:** Interaction Recurrence-severity (single episode, moderate recurrence-severity, high recurrence-severity) and state (RDS≤8/RDS>8) |
|  | 1b.3 | **T-test:** HC (RDS=4), RDS>8 (all recurrence-severities combined) |
|  | 1b.4 | Repeat of analysis 1b.3 with AD use participants removed |
| **Longitudinal analysis** (remitted; restricted to RDS≤8 for both visits)  **Imaging visits:** T0,T1 | 2a | **2(within)-x-3(between) mixed-design ANOVA**: interaction time T0/T1 - episode increase (0,1,>1)  Per identified subgroup at T0:   - 2a.1: HC - 2a.2: Single ep. - 2a.3: moderate+high |
|  | 2b | Repeat of 2a (without HC) excluding AD medicated participants   - 2a.2: Single ep. - 2a.3: moderate+high |

**Figure S5: Normative modeling using a Bayesian Generalized Linear Model on the median BOLD signal in the amygdala as a function of age, stratified by sex. A mean value for head motion of 1.3 mm was used in this model plot.**


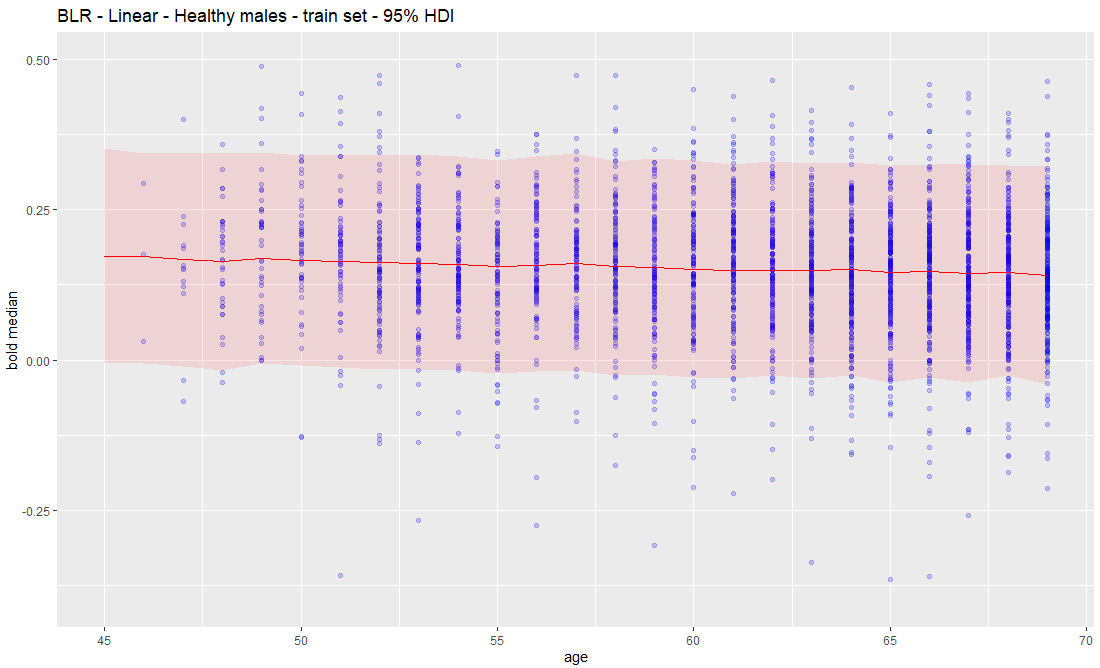


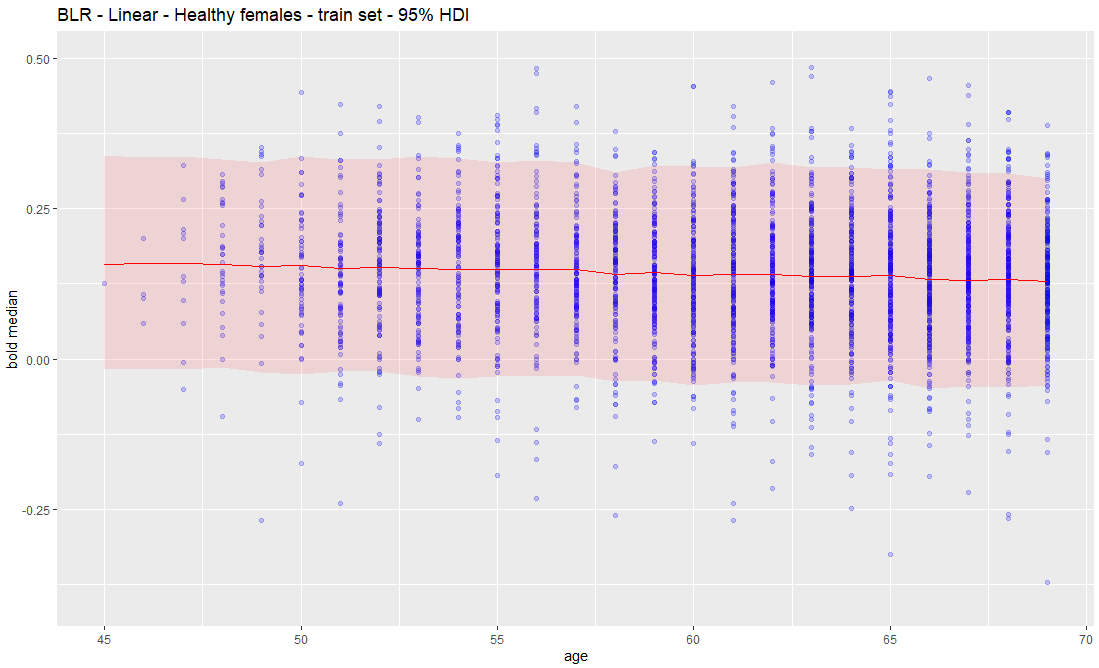


**Figure S6: Normative modeling using a Bayesian cubic B-splines Model on the median BOLD signal in the amygdala as a function of age, stratified by sex. A mean value for head motion of 1.3 mm was used in this model plot.**
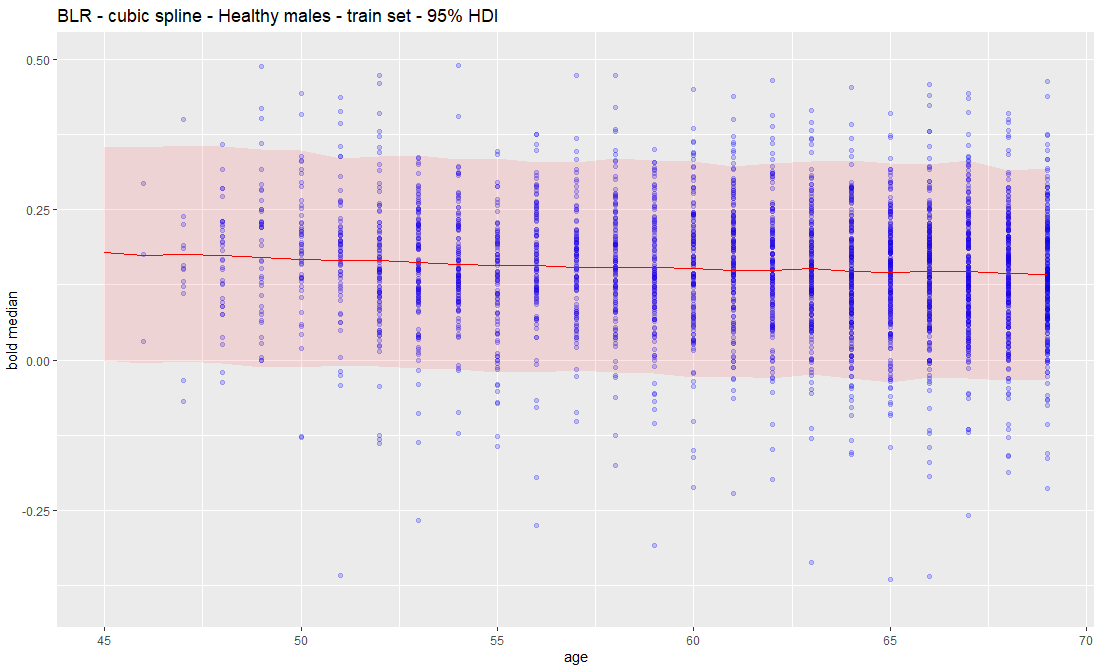

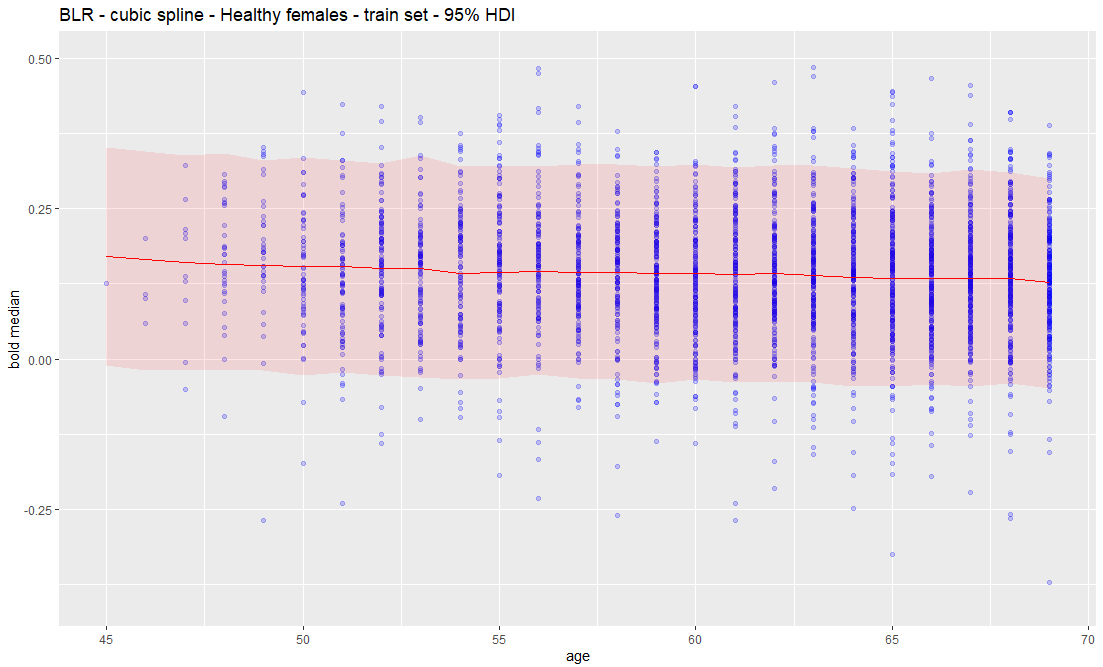


**Figure S7: Normative modeling using a Bayesian Generalized Linear Model on the 90th percentile BOLD signal in the amygdala as a function of age, stratified by sex. A mean value for head motion of 1.3 mm was used in this model plot.**


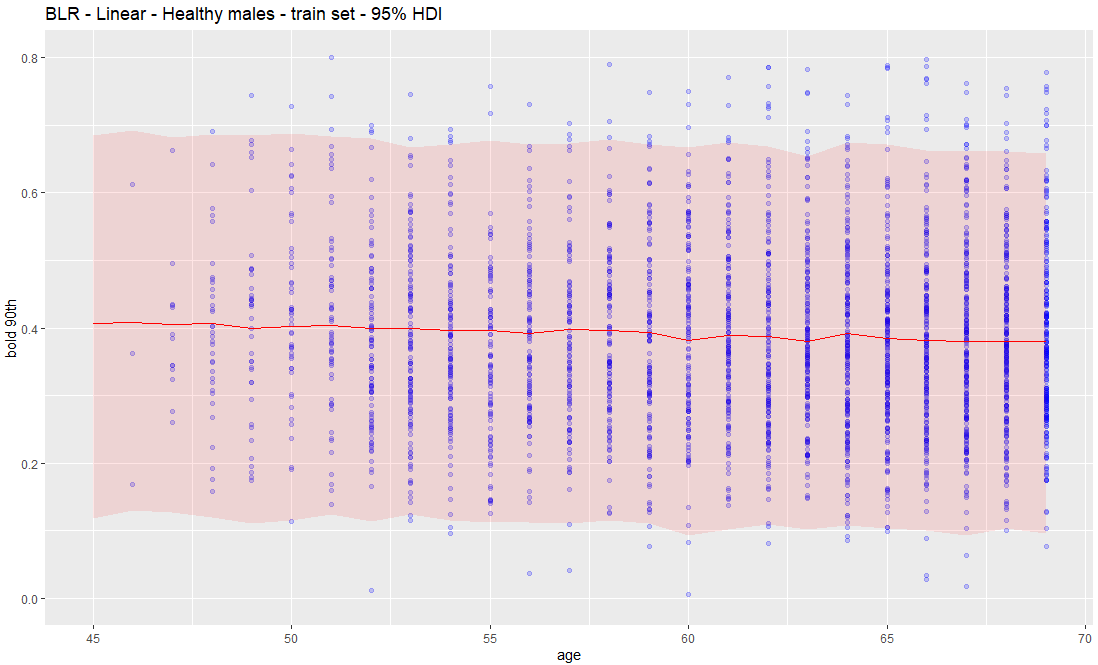


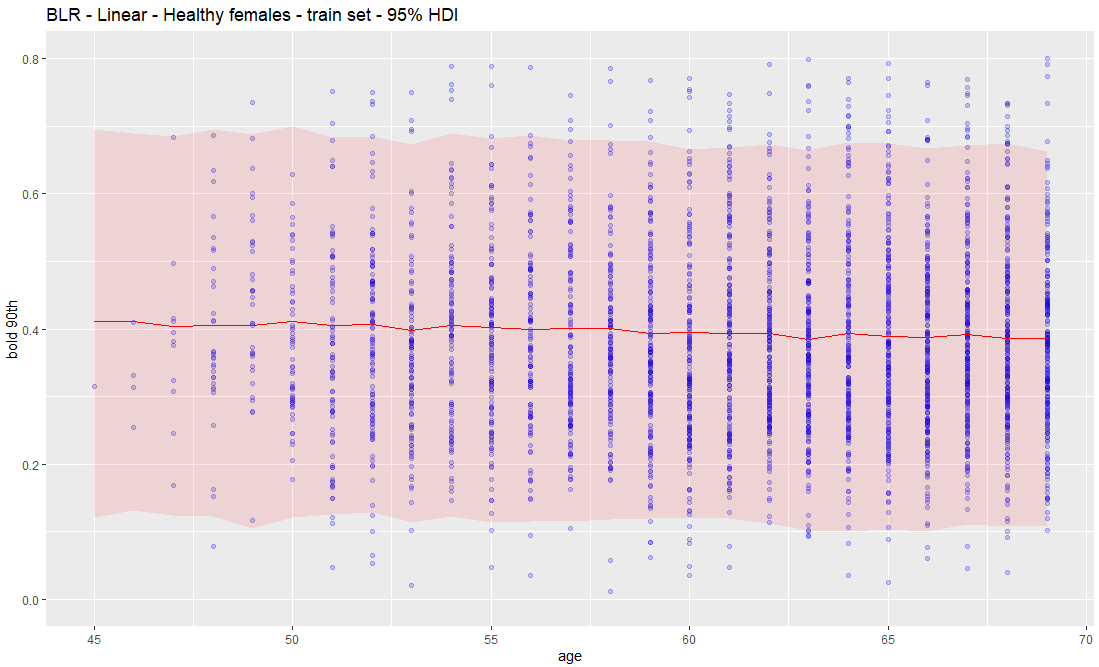


**Figure S8: Normative modeling using a Bayesian Generalized cubic B-splines Model on the 90th-percentile BOLD signal in the amygdala as a function of age, stratified by sex. A mean value for head motion of 1.3 mm was used in this model plot.**


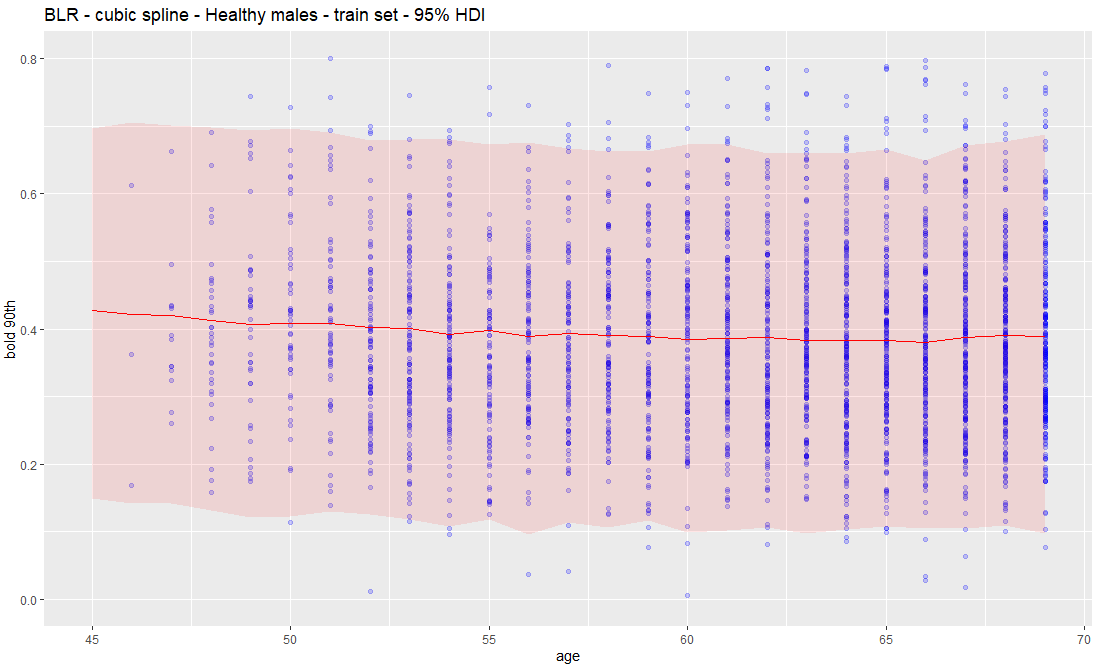

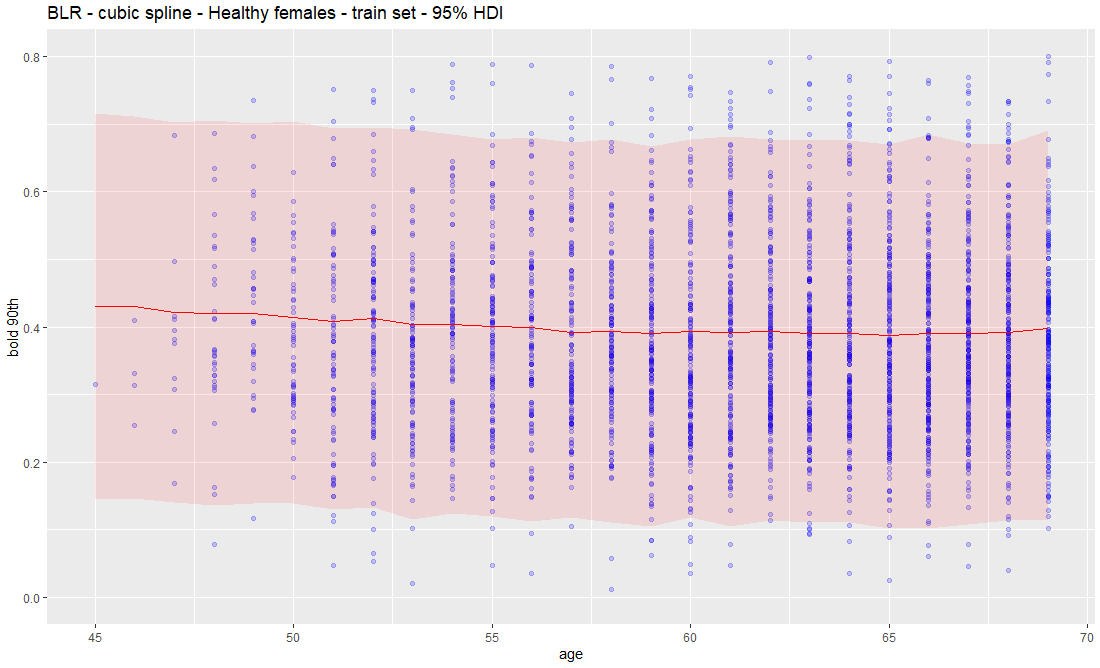


**Table S9: Bayesian Informiation Criterion (BIC) scores for linear and cubic B-splines models for BOLD Median and BOLD 90th percentile features.**

|  | **Linear model BIC score** | **B-spline model BIC score** |
| --- | --- | --- |
| **BOLD Median** | -10284 | -10252 |
| **BOLD 90th percentile** | -4366 | -4338 |

**Figure S10: Comparison of distribution of BOLD median (top) and NM-derived deviation scores of the BOLD median feature (Z-scores, bottom) (linear model used without splines).**

**
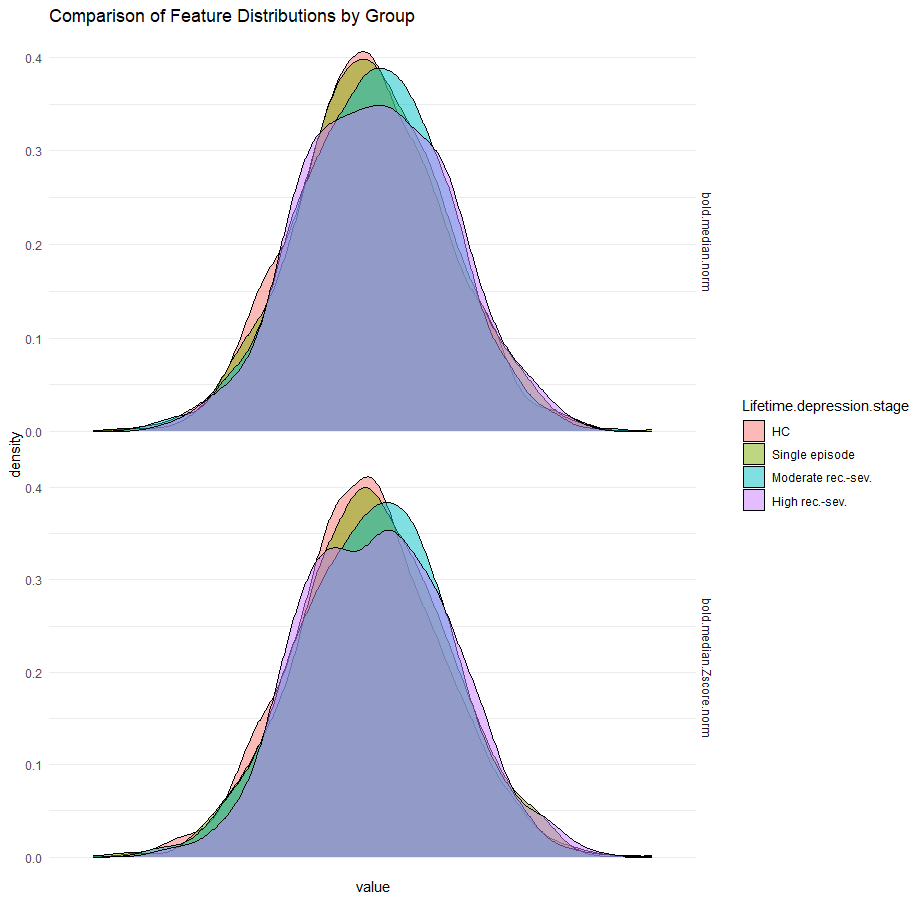
**

**Figure S11: Normative modeling using a Bayesian Generalized Linear Model on the median BOLD signal in the amygdala as a function of head motion, plotted over the used train set (all Healthy Controls)**

**
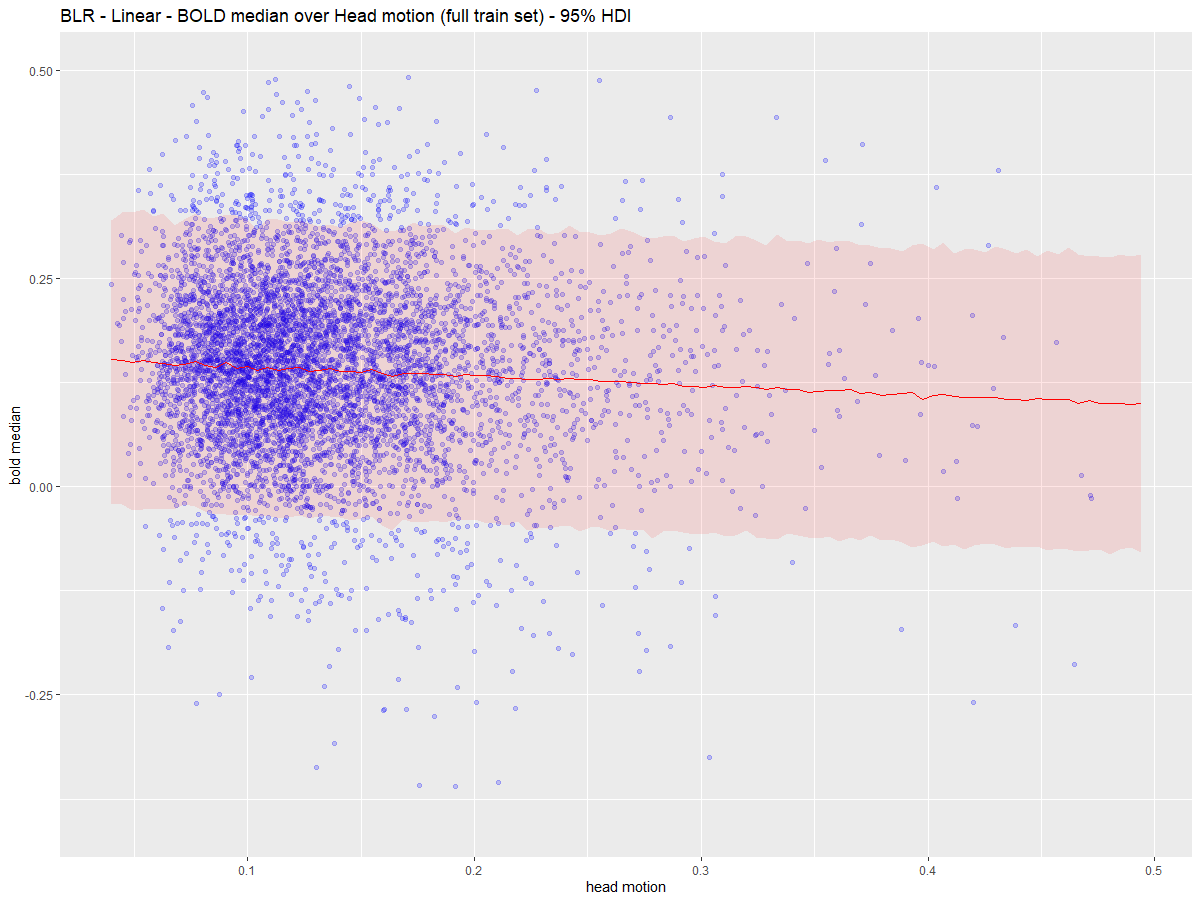
**

**Figure S12: Sensitivity analysis within the HC group to a higher ratio of repeat imaging attendees compared to the depressive subgroups**

**
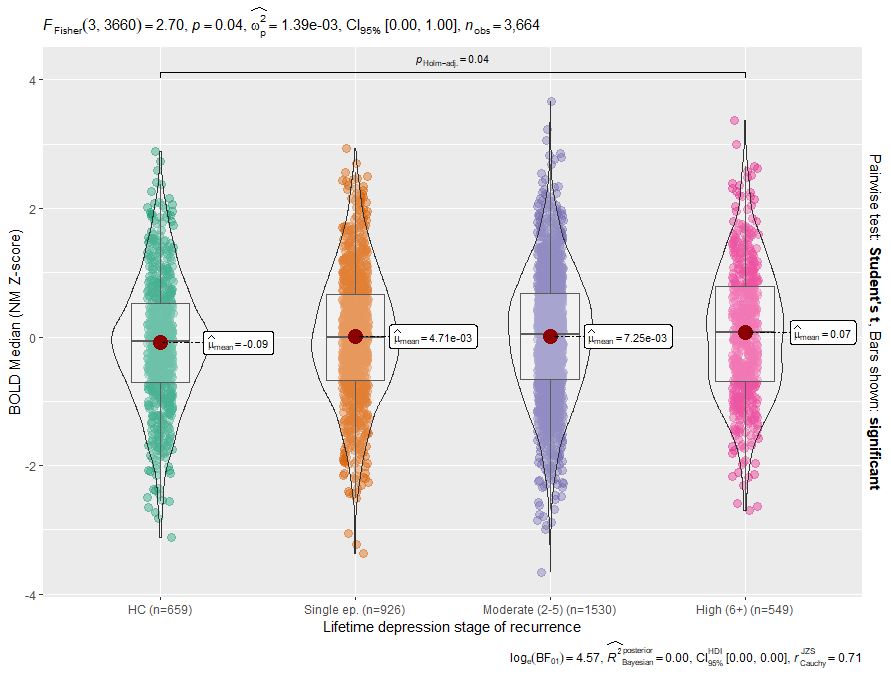
**

**Table S13: Comparison of RDS scores and repeat imaging visit ratio within the analysed subgroups**

|  | **Healthy Controls (N=1610)** | **Single episode (N=926)** | **Moderate recurrence (2-5 ep) (N=1530)** | **High recurrence (6+ ep) (N=549)** | **RDS>8 (N=277)** |
| --- | --- | --- | --- | --- | --- |
| **RDS score during imaging visit T0 (standard deviation)** | 4.66 (1.00) | 4.79 (1.08) | 5.24 (1.31) | 5.68 (1.42) | 10.19 (1.74) |
| **Repeat imaging visits** | 1037 (64.4%) | 134 (14.5%) | 184 (12.0%) | (79) (14.4%) | 31 (11.2%) |
| **RDS score during imaging visit T1 (standard deviation)** | 4.58 (0.95) | 4.82 (1.04) | 5.30 (1.30) | 5.38 (1.30) | 10.63 (2.07) |
| **Pairwise t-test of RDS score at T0-T1**  **(p-value)** | -2.08 (0.04) | -0.315 (0.75) | -0.06 (0.95) | -2.23 (0.03) | 0.61 (0.56) |

One-way ANOVA of RDS score between rMDD groups at T0 (single ep., moderate and high); F=88.2, df=2, p<0.01, ω^2^=0.05; p_Holm_<0.01 for each pairwise comparison (Single ep. - Moderate, Single ep. High, Moderate - High)

One-way ANOVA of RDS score between rMDD groups at T1 (single ep., moderate and high); F=7.42, df=2, p<0.01, ω^2^=0.03; pairwise comparisons Single ep. – Moderate p_Holm_<0.01, Single ep.-High p_Holm_<0.01, Moderate-High p_Holm_=0.66.

One-way ANOVA of the ratio of repeat imaging visits between depression-related groups (single ep., moderate and high, RDS>8); F=1.59, df=3, p=0.19.

**Figure S14: Baseline cross-sectional analysis - BOLD 90th percentile**

**
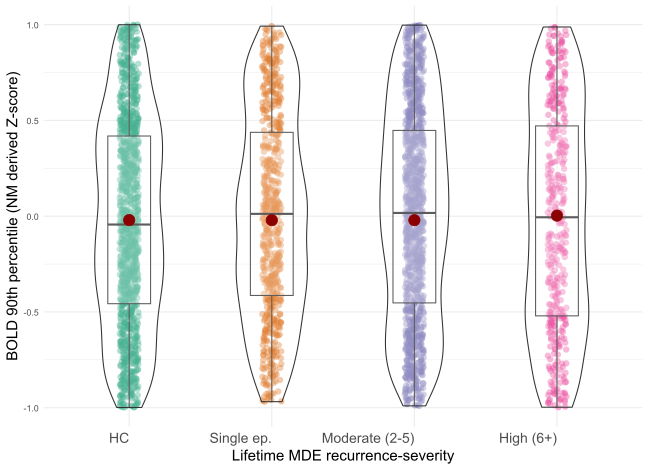
**

**Figure S15: Cross-sectional analysis - recurrence-severity moderate+high combined**

**
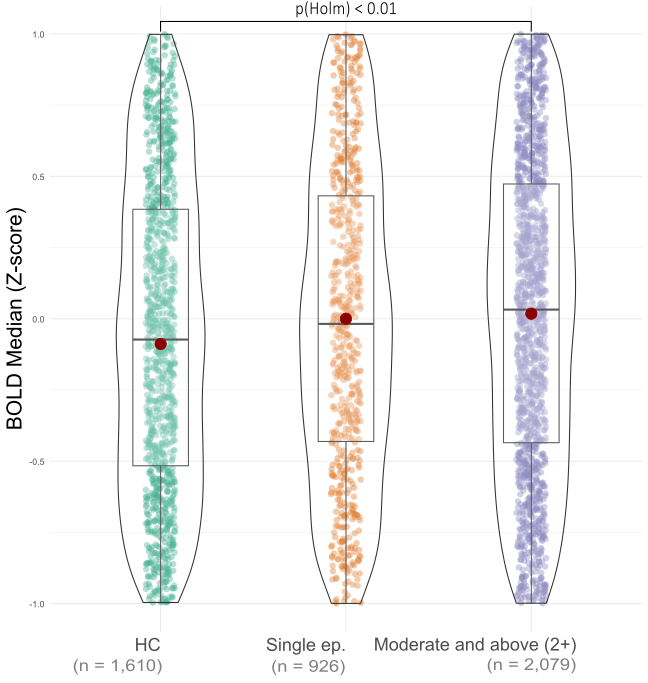
**

**Figure S16: Cross-sectional analysis - non-AD users only**

**
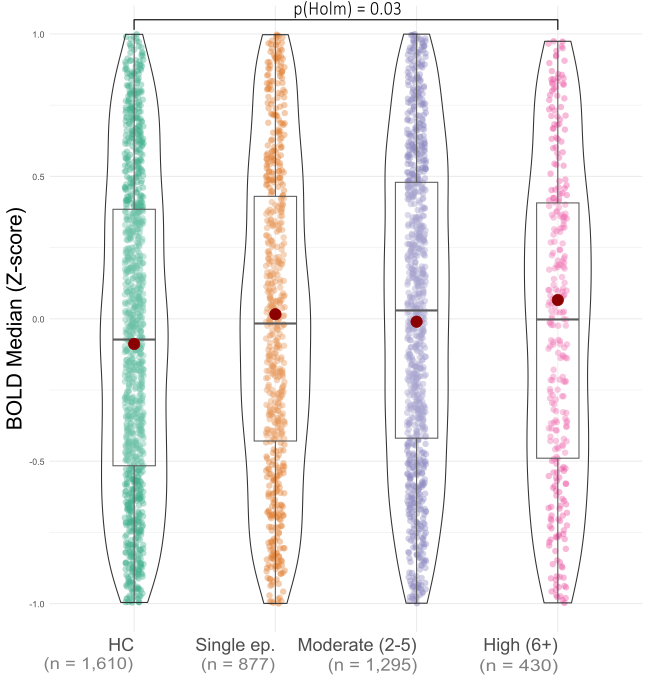
**

**S17: Cross-sectional analysis, testing for an interaction between antidepressants (AD) and recurrence-severity use, with a descriptives plot.
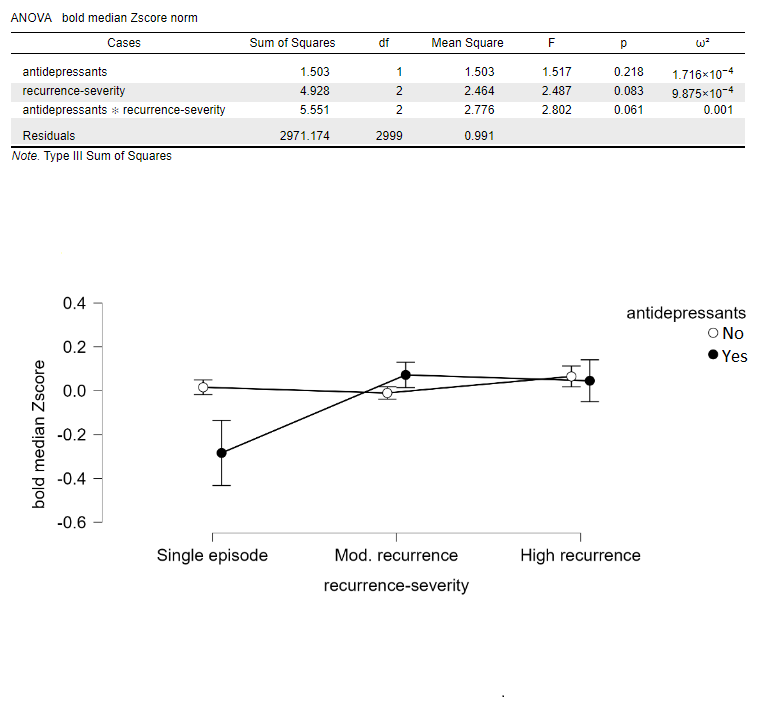
**

**Figure S18: Cross-sectional analysis: Recurrence-severity within current high symptom-severity (RDS>8)**


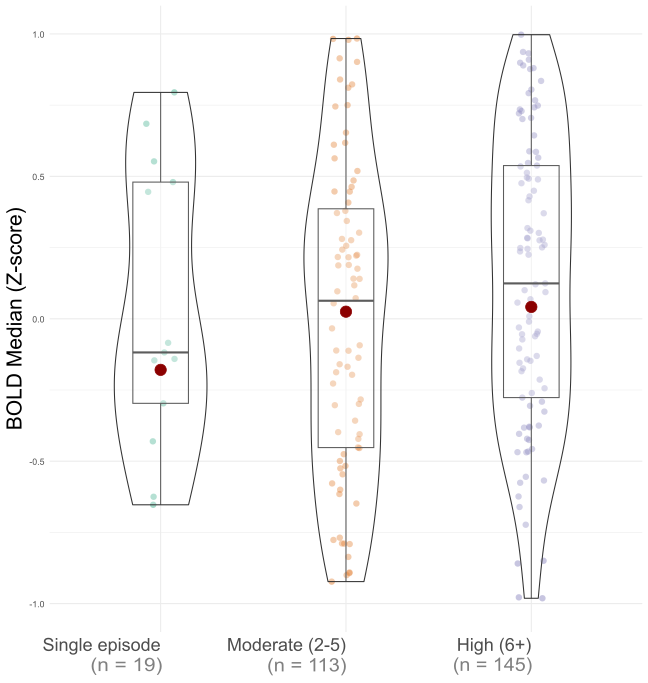


**S19: Cross-sectional analysis: Interaction recurrence-severity * state**

**
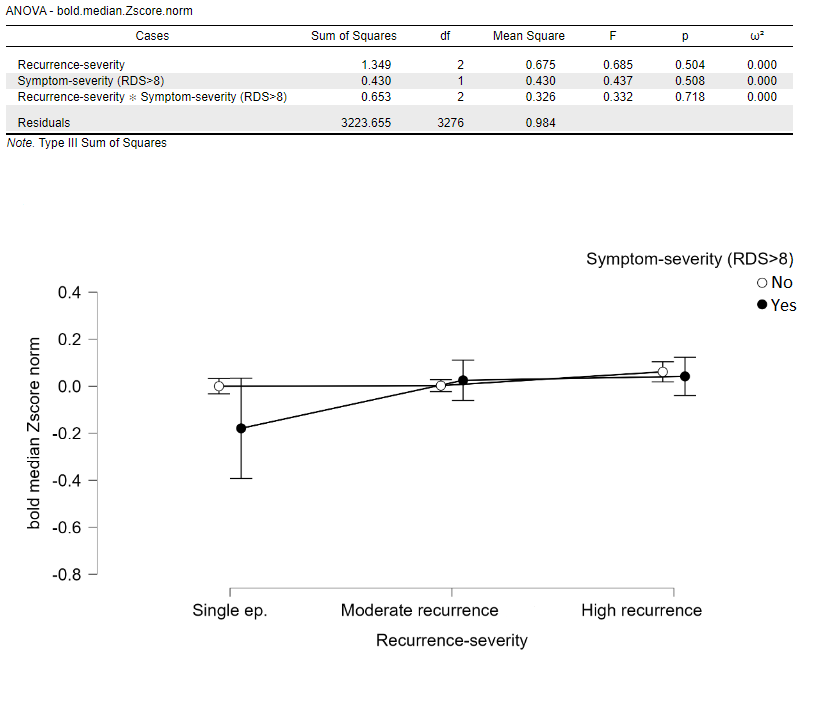
**

**Figure S20: Overview of amygdala BOLD response and current depressive symptoms (RDS=4, RDS>8, and RDS>8 excluding Antidepressant using participants) - no significant differences found**


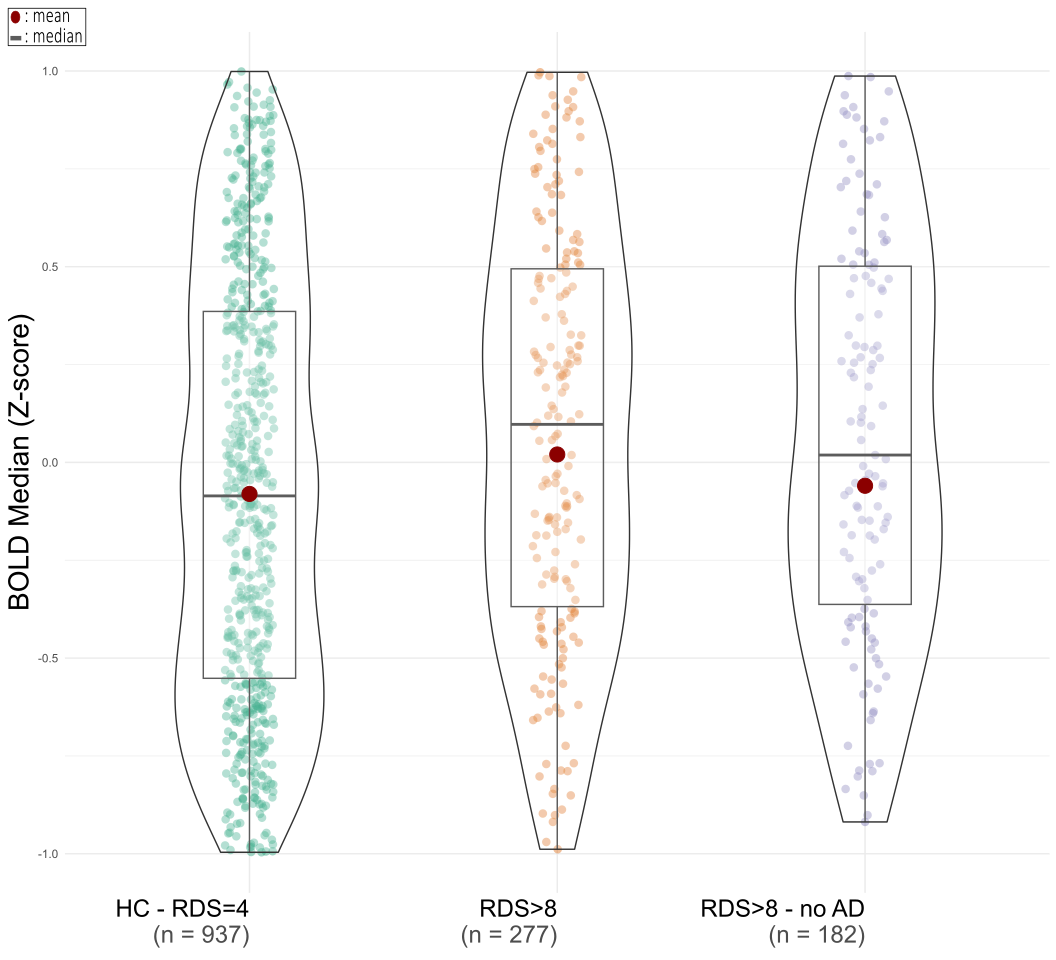


**Table S21: Sample size overview in the longitudinal analysis when excluding antidepressant (AD) medication. Time phase relates to baseline (T0) or follow-up imaging (T1), respectively.**

| **Group (T0)** | **Increase in nr. episodes (T0 to T1)** | **AD (T0 only)** | **AD (T1 only)** | **AD (T0+T1)** | **Total** |
| --- | --- | --- | --- | --- | --- |
| **Single ep.** | **0** | 0 | 1 | 1 | 2 |
|  | **1** | 0 | 0 | 0 | 0 |
|  | **>1** | 1 | 0 | 1 | 2 |
|  | **Total** | **1** | **1** | **2** | **4** |
|  |  |  |  |  |  |
| **2+ episodes** | **0** | 1 | 3 | 2 | 6 |
|  | **1** | 1 | 1 | 0 | 2 |
|  | **>1** | 3 | 7 | 4 | 14 |
|  | **Total** | **5** | **11** | **6** | **22** |

**Figure S22: Longitudinal data of the (NM-adjusted) amygdala BOLD signal, excluding antidepressant (AD) use, with episode increase between repeated visits T0 and T1 in columns (0, 1 and >1) and lifetime recurrence classification during the first visit in rows. No significant interaction between time and increase in episodes was found in either group.**


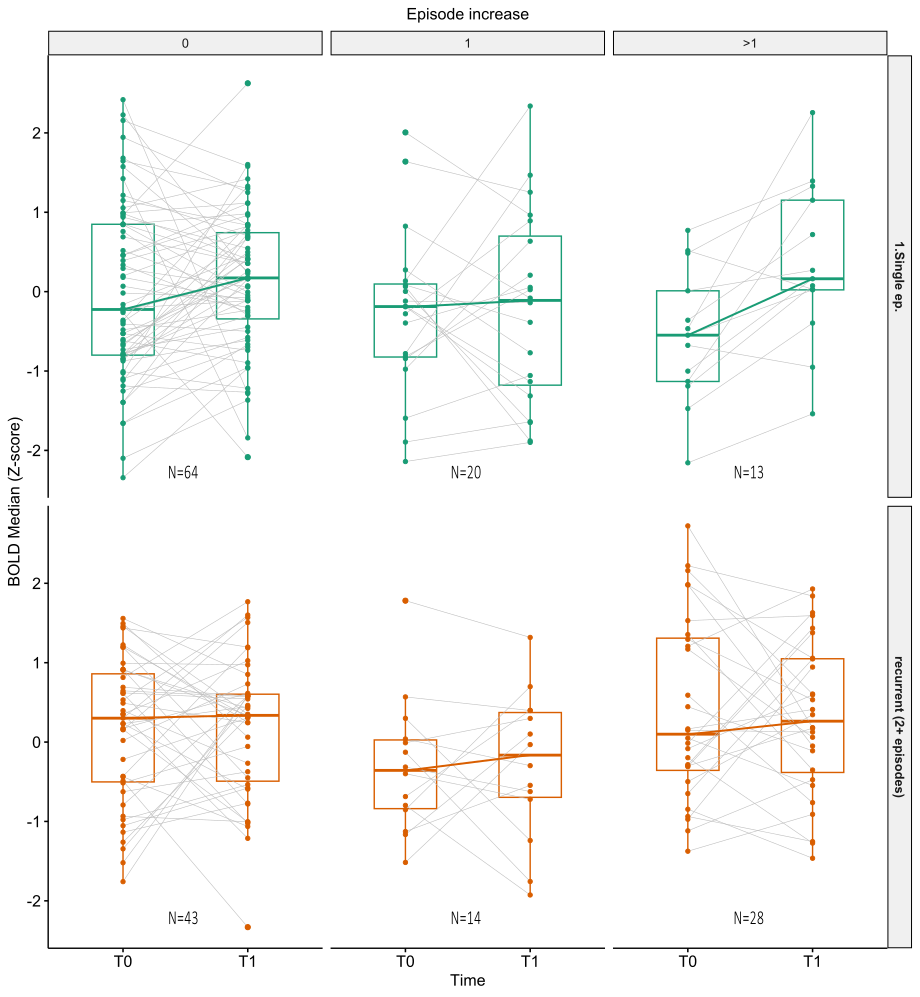

Supplement: Van Den Berg et al. supplementary material [file S0033291725101797sup001.docx]
